# An Overview of Potential Targets for Treating Amyotrophic Lateral Sclerosis and Huntington's Disease

**DOI:** 10.1155/2015/198612

**Published:** 2015-07-29

**Authors:** Caroline Zocatelli de Paula, Bruno Daniel Correia Gonçalves, Luciene Bruno Vieira

**Affiliations:** Laboratório de Neurofarmacologia, Departamento de Farmacologia, Instituto de Ciências Biológicas (ICB), Universidade Federal de Minas Gerais, Campus Pampulha, 31270901 Belo Horizonte, MG, Brazil

## Abstract

Neurodegenerative diseases affect millions of people worldwide. Progressive damage or loss of neurons, neurodegeneration, has severe consequences on the mental and physical health of a patient. Despite all efforts by scientific community, there is currently no cure or manner to slow degeneration progression. We review some treatments that attempt to prevent the progress of some of major neurodegenerative diseases: Amyotrophic Lateral Sclerosis and Huntington's disease.

## 1. Introduction

Neurodegenerative diseases are characterized by a slow and progressive loss of neurons in different areas of the central nervous system. Motor neurons are impaired in Amyotrophic Lateral Sclerosis (ALS) as well as thorny striatal neurons in Huntington's disease (HD) [1]. ALS symptoms include a progressive muscle phenotype of spasticity, hyperreflexia, fasciculations, atrophy, and paralysis. The disease is usually lethal within 3–5 years after the diagnosis and in most cases, patients die from respiratory failure [1]. The causes of the disease and the mechanisms causing premature death of motor neurons are not clear. In addition, two types of the disorder are recognized, familial ALS (fALS) and the most common sporadic ALS (sALS) [1].

Currently the only available treatment for ALS is Riluzole, approved for use in 1995. However, Riluzole only slows down the progression of the disease and gives the patient an average of 3 months of extended life span [2]. The exact mechanism of action of Riluzole is unknown; several papers have demonstrated its inhibition of sodium, calcium, potassium, and glutamate currents [3]. The coadministration of Riluzole with other potential therapeutic agents is constantly being assessed and is usually with no promising positive results [4, 5]. Therefore, the safety of the combination of Riluzole with a second study drug is unpredictable.

Huntington's disease (HD) is an inherited neurodegenerative disorder, characterized by progressive loss of neurons in the whole brain, notably in the basal ganglia and cerebral cortex, resulting in worsening chorea, cognitive and psychiatric disturbances [6]. In the United States, HD occurs in about 1 in 10,000 people. Currently about 30,000 people in the US have HD and up to 200,000 are at risk [7]. HD is caused by expanded polyglutamine cytosine-adenine-guanine (CAG) repeat sequence in the autosomal dominant gene that encodes huntingtin (Htt, 4p16.3) [8]. Mutant huntingtin protein accumulates and produces transcriptional dysregulation, proteasomal, autophagical, mitochondrial, and metabolic dysfunctions, oxidative stress, apoptosis, neuroinflammation, and consequent neurodegeneration, especially in the striatum [8, 9]. Unlike ALS, no major treatment has been developed specifically for Huntington's disease. Some symptomatic treatments are available for some motor or psychiatric features [10].

Since both ALS and HD are neurodegenerative disorders, the neuronal loss is progressive and leads to severe disability and reduced quality of life and life expectancy [11]. Although multiple factors are involved in the pathogenesis of these neurodegenerative diseases, which have heterogeneous etiology and background, the mechanisms underlying neuronal death are similar, enabling development of drugs that target many disorders [12]. Nervous system damage in response to an insult may lead to acute or delayed neuronal death, apoptotic cell death, neuronal degeneration, injury and loss, and gliosis [11]. Thus, neuroprotection can be an important therapeutic intervention that prevents the death of vulnerable neurons and also retracts progression of the disease.

The aim of this review was to discuss possibilities of multimodal and neuroprotective therapies for ALS and HD, employing currently available drugs and potential targets that might be exploited in the future.

## 2. Amyotrophic Lateral Sclerosis (ALS)

Possibly because the pathogenesis of ALS remains mostly unknown, development of treatments that are effective across the spectrum of sporadic and familial ALS has not been achieved. Considerable evidence supports the involvement of mitochondrial dysfunction and oxidative stress, autophagy, apoptosis, and protein aggregation in the pathogenesis of ALS [13]. Several exciting potential targets for drug intervention to lessen neurodegeneration in ALS are in development [14–16]. We described some of the promising candidates for treating ALS.

### 2.1. Mitochondrial Function and Oxidative Stress Pathways

The involvement of mitochondria in oxidative damage, calcium buffering, and signaling of apoptotic pathways puts the organelle in perspective for therapeutic approaches. Questions such as whether mitochondrial dysfunctions are a trigger or a consequence in neurodegenerative disease dynamics are still in debate, and increasing interest and discoveries are in course regarding mitochondrial modulators [17]. Studies in ALS patients present strong evidences of mitochondrial dysfunction [18–20]. Moreover, mutant SOD1 mouse model for ALS shows morphological alterations in motor neurons and skeletal muscle tissue before onset of neurodegeneration symptoms, and similar abnormalities are found in sporadic ALS patients [21–23]. Furthermore mitochondrial alterations are not restricted to SOD1 mutants as demonstrated in TDP-43 mutant models [24].

MitoQ is a mitochondrial antioxidant that contains the antioxidant Quinone linked to a lipophilic triphenylphosphonium cation. Researchers showed that MitoQ prolonged life span and the pathology of SOD-knockout* Drosophila melanogaster*, the enzyme involved in ALS [25]. In addition, the compound has been shown to exert neuroprotective effects in SODG93A mice, slowing functional decline, decreasing oxidative damage and disease progression, and increasing survival [26]. Furthermore, other studies showed that MitoQ preincubation prevented the cell death observed in cultures of motor neurons + SOD-mutant astrocytes alone [27].

### 2.2. Melatonin

Melatonin is a natural hormone produced and secreted by the pineal gland. It is currently used to increase sleep efficiency and improve cardiovascular system and as an antiaging drug [28, 29]. Recent researches in experimental models showed positive effects of melatonin in major neurodegenerative disorders such as ALS, Parkinson's disease, Alzheimer's disease, and HD [30]. In ALS, the most promising effects of melatonin are those of blocking apoptotic pathways and reducing oxidative damage. The mechanisms of melatonin antiapoptotic effects are not completely clear, although the mitochondria have been identified as its target [31].

Oxidative damage caused by free-radicals is accepted as a common link between neurodegenerative diseases and melatonin acts scavenging these free-radicals showing antioxidant activity [32]. Experiments demonstrated attenuation of superoxide induced cell death and the modulation of glutamate toxicity by melatonin* in vitro* NSC-34 motor neurons culture [33].

Other recent study showed an inhibitory activity of melatonin over apoptotic caspase 1/cytochrome c/caspase 3 apoptotic pathway, with melatonin preventing cell death. In ALS SODG93A mice, melatonin inhibited motor neuron death in the ventral horn of spinal cord and delayed disease onset and mortality [34]. The hormone also inhibited Rip2/caspase 1 pathway. In addition, the work had novel findings in showing an association between disease progression and loss of melatonin and melatonin 1A receptors (MT1) in spinal cord of mice. These latter are interesting since MT1 receptors are linked to other major neurodegenerative diseases such as HD and Alzheimer's disease [35–37].

Melatonin clinical trials were so far aimed mostly at insomnia and sleep disturbances [38]. However more recently reduced oxidative damage was reported in patients treated with enteral melatonin at high-doses [33]. Melatonin showed well-toleration event at high-doses and crosses easily the blood-brain barrier further supporting its therapeutic potential.

### 2.3. Protein Aggregation, Altered Autophagy

One of the consequences of the genetic mutations involved in neurodegenerative diseases is the appearance of misfolded mutant protein aggregates [39]. These aggregates can be toxic and can affect organelles such as mitochondria by causing membrane disruption. In this way, some studies support that clearance of these aggregates could be a therapeutic intervention. In ALS, a link between the disease and protein aggregation altered autophagy was initially observed on morphological studies of spinal cord tissues of ALS patients and models, showing increased number of autophagosomes [40–42].

In this way, as evidences aroused that impaired autophagy may play a pathogenic role in neurodegeneration, compounds promoting autophagy started to be tested. Progesterone and trehalose, two autophagy stimulators, have showed promising results in the SOD model, delayed disease onset and prolonged survival, all related to an induction of the autophagic flux [43, 44].

Rapamycin, an FDA approved drug, that induces autophagy was tested in the SOD1-G93A model with negative results, enhancing the rate of disease progression and motor neuron death [45, 46]. Thus Rapamycin may be, in part, detrimental in ALS due to its immunosuppressive action [45]. However, there are recent data showing that Rapamycin enhances autophagy in immunodeficient mice avoiding the progression of ALS [47]. These authors used a strategy to avoid the immunosuppressive effect of Rapamycin and so observed its action on increasing autophagy. Then in this case Rapamycin slowed down the progression of ALS [47].

In addition to the previously mentioned effects, the hormone melatonin has also demonstrated neuroprotective activity through enhanced autophagy [48, 49]. Melatonin protected against apoptosis via a mitochondrial pathway, reducing caspase 3 activity, cytochrome c release, and increasing LC3-II/LC3-I levels, an autophagy marker, as demonstrated by Chen et al. [48]. The granular corneal dystrophy type 2 hallmark is linked to impaired autophagic degradation of mutant protein deposits [49]. Choi et al. also demonstrated that melatonin activates autophagy via the mammalian target of Rapamycin pathway (mTOR). Melatonin has showed positive effects in autophagosome formation and maturation, and its cotreatment with Rapamycin had additive effects in autophagic clearance of mutant protein aggregates, in comparison to either drug alone [49]. Although Rapamycin effects of suppression of protective immune responses and enhancement of protective autophagy can cancel each other out in general health improvement [45], these findings suggest that more specific targeting of autophagy with drugs that produce less side effects holds potential for halting disease and neurodegeneration. For that matter melatonin treatment can be a more efficient option with fewer side effects. Moreover it could be possible that cotreatment with melatonin and lower dosages of Rapamycin would increase efficacy while maintaining Rapamycin-induced immunosuppression controlled.

### 2.4. Targeting the Endocannabinoid System

The endocannabinoid system is recognized as playing a major role in modulating various processes in the body. Increasing evidences suggest an antioxidant, anti-inflammatory, neuroprotective, and other activities such as improving appetite, anxiety, and depression related to endocannabinoids and cannabinoid therapies [50]. However, there are still major controversies regarding therapeutical applications of the endocannabinoid system, such as whether therapeutic effects are achieved by direct agonism/antagonism of cannabinoid receptors or by modulating the endocannabinoid system tonus, by reducing degradation of natural cannabinoids such as anandamide [50, 51].

In ALS, the antioxidant, anti-inflammatory, and neuroprotective activities of cannabinoids are expected to improve ALS symptoms. First surveys assessed marihuana usage in ALS patients and associated it with improvements of appetite, depression, pain, spasticity, and drooling [52].

In ALS SOD1-G93A mice, scientists assessed the effects of cannabinol, a nonpsychotropic compound of the plant. Positive effects regarding disease progression and delay of approximately 2 weeks of disease onset were observed, providing evidence of cannabinoid treatment for ALS [53]. Further evidence was found, showing that cannabinol, another phytocannabinoid, also exerts positive effects of normalizing mitochondrial dynamics associated with caspase 3, DNM1L, and synaptophysin levels in models [54]. More recently, a combination of phytocannabinoids called Sativex was tested in SOD1-G93A mice with moderately positive results [55]. All of these emerging findings point towards neuroprotective and antiapoptotic activities of the cannabinoids in neurodegenerative processes. Better results are expected by the design of more specific compounds, acting on specific populations of cannabinoid receptors, as suggested by discoveries of specific neuroprotective population of cannabinoid receptors [56].

## 3. Huntington's Disease

A neuropathological hallmark of Huntington's disease is the presence of neuronal nuclear inclusions and cytoplasmic aggregates of misfolded mutant huntingtin protein (mHtt) [9]. The mHtt accumulates and produces transcriptional dysregulation, proteasomal, autophageal, mitochondrial, and metabolic dysfunctions, oxidative stress, apoptosis, neuroinflammation, excitotoxicity, and consequent neurodegeneration [8, 9]. As HD is a genetic disease, affected patients have abnormal huntingtin from the very first moment of the protein's expression, which suggests that neuronal abnormalities might be present since the start. Targeting these pathways might be a cleaver strategy for treating HD.

### 3.1. Apoptotic Pathways

Neuronal cell death in HD is associated with neuronal apoptosis, particularly with the initiation of the intrinsic mitochondrial apoptotic pathway [12]. Markers for apoptotic cell death are activated in striatal neurons from both patients with HD and animals models [57]. Activation of caspases 3 and 9 and release of cytochrome c from the mitochondria into the cytosol are observed both in the brains of patients and animals with HD [58].

Minocycline is an antibiotic, reported to exert neuroprotective activities through caspase 1, caspase 3, inducible form of nitric oxide synthase, and p38 mitogen-activated kinase (MAPK). It has good oral bioavailability, tolerability, and crosses blood-brain barrier easily. In ALS models, minocycline delayed disease onset and extended survival. It also inhibited cytochrome c release, which was demonstrated both* in vivo* and in isolated mitochondria [59].

Chen et al. reported that the inhibition of caspases 1 and 3 expression by minocycline delayed mortality in R6/2 mice, a model of HD. In addition, minocycline attenuated dopaminergic cell loss and delayed mortality in MPTP-treated mice. Minocycline was taken to human trials for HD as a promising therapy but failed. Despite its promisor results, it has encountered limitations for its use due toxicity in pharmacological dosages [59]. Moreover, the currently available caspase inhibitors are toxic in pharmacological doses, precluding their immediate use in human studies [12].

### 3.2. Oxidative Stress

Oxidative stress is characterized by an imbalance between reactive oxygen species (ROS) and antioxidant systems [60]. Interestingly, Chen et al. described a correlation between lipid peroxidation products in plasma and degree of severity in patients with HD [59]. Klepac et al. also reported an occurrence of oxidative stress in HD [61].

Dimethyl fumarate (DMF), an essential member of fumaric acid ester (FAE) family, is the active ingredient of BG-12, which has been offered as an effective oral treatment option for patients with relapsing remitting multiple sclerosis (RRMS) [62]. DMF can activate transcription factor nuclear factor (erythroid-derived 2)-related factor 2 (Nrf2). Nrf2 plays an important role of anti-oxidative pathways for tissue protection [63]. There is compelling evidence of the disruption of the Nrf2 system in HD and the contribution of Nrf2 activation to ameliorating oxidative stress and mitochondrial dysfunction in neuronal tissue damage in HD. Jin and colleagues have shown that mHtt disrupts Nrf2 signaling, which contributes to impaired mitochondrial dynamics and may enhance susceptibility to oxidative stress in STHdh (Q111/Q111) cells, striatal cells expressing mHtt [64]. Moreover, DMF treatment leads to an increase in Nrf2 staining in neuronal subpopulations relevant for motor functions, concomitant with elevated Nrf2 immunoreactivity in R6/2 mice, which mimic many aspects of HD. Additional studies in N171-82Q mice, another transgenic mouse model of HD, showed that 2-cyano-3,12-dioxooleana-1,9-dien-28-oic acid-ethyl amide (CDDO-EA) and 2-cyano-3,12-dioxooleana-1,9-dien-28-oic acid-trifluoroethyl amide (CDDO-TFEA) upregulate Nrf2/ARE induced genes in the brain and peripheral tissues and reduce oxidative stress, improving motor impairment and increasing longevity [65].

### 3.3. Neuroprotective Cell Signaling Pathways

The signaling pathways involved in HD are not yet clearly elucidated [66]. Thus, it is possible that alterations of receptor-mediated signaling pathways could contribute to protection or exacerbation of cell death cascades in the symptomatic and/or presymptomatic phases of HD [67]. Recently, Doria et al., 2015, described the effect of a positive allosteric modulator (PAM) for metabotropic glutamatergic receptor type 5 (mGluR5), named CDPPB (3-cyano-N-(1,3-diphenyl-1H-pyrazol-5-yl)benzamide) [68]. This drug was capable of delaying some symptoms related to HD [68, 69]. Chronic treatment of BACHD mice (model of HD) with CDPPB 1.5 mg/kg for 18 weeks increased the activation of cell signaling pathways, including increased AKT and ERK1/2 phosphorylation, and augmented the BDNF mRNA expression. CDPPB chronic treatment was also able to prevent the neuronal cell loss that takes place in the striatum of BACHD mice and decrease mutant huntingtin aggregate formation [68]. Moreover, CDPPB chronic treatment was efficient to partially ameliorate motor incoordination and to rescue the memory deficit exhibited by BACHD mice. Importantly, no toxic effects or stereotypical behavior was observed upon CDPPB chronic treatment; however the exact CDPPB mechanism of action and more safety tests need to be performed [68].

### 3.4. Excitotoxicity

Excitotoxicity is known to be an important piece in the development of HD. Thus, antiglutamatergic agents may, therefore, have beneficial neuroprotective effects [70]. One of these agents is tryptophan metabolite kynurenic acid (KYNA), which is an endogenous NMDA receptor antagonist [70]. Although the neuroprotective KYNA shows unaltered levels in mice models, the significant elevation in the concentrations of neurotoxic kynurenine pathway compounds leads to a shift in the metabolism resulting in relative KYNA deficiency [70]. These findings raise the possibility that increasing KYNA effect would be beneficial from a therapeutic aspect. However, higher doses of KYNA have low solubility and poor penetration to blood-brain barrier [71].

Memantine is a noncompetitive N-methyl-D-aspartate (NMDA) receptor antagonist that stabilizes glutamatergic tone. Memantine can attenuate the excitotoxic mechanism [72]. One interesting study examined Memantine as a treatment for patients suffering from Huntington disease. The administration of 20 mg of Memantine/daily significantly improved motor symptoms and improved chorea but failed to improve patient's cognitive, behavioral, functional, or independence ratings [73].

## 4. Conclusion

Neurodegenerative diseases are multifactorial and despite recent progress, basic needs such as the definition of disease biomarkers and molecular mechanisms of neurodegeneration are still to be addressed. No major treatment has been developed specifically for ALS and HD. Some symptomatic treatments are available for some motor or psychiatric features. In the meantime, the revelation of new mechanisms involved in the disease onset and progression gives opportunity for novel approaches in symptomatic treatment. The development or improvement of disease models is also necessary for better assessment of drugs and pathological mechanisms. Multifunctional and multitarget approaches will probably be needed to restore neuronal health when cell dysfunction is present. We must have in mind that multitargeted and combined therapies may be an option in that regard.

## Review Criteria

Articles were selected based on searches of PubMed using a number of different search terms, such as “Neurodegenerative diseases” “Huntington's disease,” “Amyotrophic Lateral Sclerosis,” “neuroprotection,” “neurodegeneration,” “pathogenesis,” “mitochondria,” “apoptosis,” “huntingtin,” and “autophagy.” Only full-text papers written in English and its references were selected.
